# Receiver Operating Characteristic curve analysis determines association of individual potato foliage volatiles with onion thrips preference, cultivar and plant age

**DOI:** 10.1371/journal.pone.0181831

**Published:** 2017-07-26

**Authors:** Calum R. Wilson, Noel W. Davies, Ross Corkrey, Annabel J. Wilson, Alison M. Mathews, Guy C. Westmore

**Affiliations:** 1 Tasmanian Institute of Agriculture (TIA), New Town Research Laboratories, University of Tasmania (UTAS), New Town, Tasmania, Australia; 2 Central Science Laboratory, UTAS, Hobart, Tasmania, Australia; University of Idaho, UNITED STATES

## Abstract

Tomato spotted wilt virus (TSWV) causes sporadic but serious disease in Australian potato crops. TSWV is naturally spread to potato by thrips of which *Thrips tabaci* is the most important. Prior studies indicated possible non-preference of potato cultivars to *T*. *tabaci*. Select potato cultivars were assessed for non-preference to *T*. *tabaci* in paired and group choice trials. Cultivars ‘Bismark’, ‘Tasman’ and ‘King Edward’ were less preferred than ‘Atlantic’, ‘Russet Burbank’ and ‘Shepody’. Green leaf volatiles were sampled using solid-phase microextraction from the headspace of potato cultivars of two ages that differed in *T*. *tabaci* preference. Analysis of headspace volatile data using Receiver Operating Characteristic curves identified individual volatiles associated with *T*. *tabaci* preference and non-preference, young and old plants and individual cultivars. These data could be used to inform breeding programs for selection of *T*. *tabaci* resistance to assist with TSWV management, and biological testing of novel thrips management compounds.

## Introduction

Virus diseases pose significant threats to productivity of most crop plants [1]. Potato (*Solanum tuberosum* L.), the world’s third largest staple food crop after wheat and rice, suffers from several virus diseases, exacerbated by vegetative propagation which can efficiently transfer virus infections between generations [2]. In potato, infections with Tomato spotted wilt virus (TSWV) are generally rare [3], however in Australia, major epidemics can occur which may lead to complete crop loss [4–6]. TSWV is naturally spread by certain thrips species and in Australia the most important for transmission of TSWV in potato is onion thrips (*Thrips tabaci* Lindeman) [5]. Prior observations comparing potato cultivars for resistance to TSWV revealed that in field exposure trials, cv. ‘Bismark’ had significantly lower incidence of TSWV infection, and reduced thrips feeding scars [7]. In contrast when mechanically inoculated with TSWV ‘Bismark’ readily succumbed to infection. This suggested the field resistance observed may relate to thrips vector non-preference rather than resistance to virus infection [7]. The absence of effective virus-resistant cultivars means thrips resistance is an attractive option for TSWV management avoiding interactions between vector and host plant. However, breeding crops for control of virus spread through resistance to vector species can be challenging. Resistant crops can be selected that restrict the colonisation and multiplication of the vector insect, providing effective reduction in losses due to pest feeding damage [8]. This may not however be sufficient to reduce virus acquisition or transmission. Indeed plants that do not readily support colonisation of virus vector species can be efficiently infected by a non-persistently transmitted virus through transient feeding [9].

Virus diseases in potato are commonly managed by ensuring seed tubers have low virus incidence through seed certification protocols [2, 10], rogueing of infected plants within the crop, removal of volunteer potatoes and alternate weed hosts from the crop vicinity, and control of major insect vectors, primarily through strategic insecticide application [1, 2] However with TSWV epidemics, the majority of inoculum comes from sources external to the crop, and as such seed certification plays a reduced role in disease management. The use of resistant cultivars can provide another important avenue for virus disease control [11, 12]. However, with the sporadic and limited global distribution of the disease in potato, TSWV resistance has not been a breeding priority for the major potato breeding programs. Most plant breeders looking to combat virus diseases focus on selection and introgression of major resistance genes that target the virus pathogen. However, no major TSWV resistance gene has been identified in potato. Furthermore TSWV has many variant strains and a propensity for breaking resistance in crops where resistance genes are available [13, 14]. Alternatively, as many plant viruses, including TSWV, rely on an invertebrate vector species to facilitate spread between plants, targeting host genes that affect attraction, settling, feeding and fecundity of these vectors can be a valuable tool in virus disease management.

Mechanisms of host plant resistance to insects has been defined as non-preference, antibiosis or tolerance [15]. Non-preference is the most effective resistance form when dealing with virus vectors, as this reduces host plant contact by the vector, and diminishes opportunities for virus transmission events [16]. Non-preference can occur through interference with location of the plants, by altering colour or growth habit, or disrupting initial settling through leaf attributes such as waxes, trichomes, ridges and volatiles [16]. The role of plant volatiles in either attracting or repelling insects to plants is well documented [17]. For thrips, like most phytophagous insects, olfactory cues are important in the insect’s initial orientation to a plant [18]. Insects can be both attracted or repelled by plant volatiles [19–21]. Attractants can assist in locating food sources, potential mates, and may be used to identify oviposition sites [22, 23]. Repellent plant volatiles provide insects with olfactory cues of the presence of non-hosts or harmful substances [24]. These have the potential for use as new tools for crop protection as repellent crop treatments [25]. Identification of the quantity and quality of volatiles involved in plant-insect interactions is the first step toward developing such strategies that manipulated host-finding behaviour [26]. In a meta-analysis of plant herbivore interactions with plant volatiles, Szendrei and Roderigues-Soaona (2010) [26] found 76% of all characterised interactions involved attraction of the herbivore, with only 3% of the studies testing compounds that act as repellents. They conclude that the application of plant volatiles as repellents is an underexplored area.

Potato foliage continually releases volatile compounds into the air, and analysis of volatile organic compounds (VOCs) emitted from potato green foliage has been the subject of several prior studies. Potato foliage VOCs are dominated by an abundance of sesquiterpenes and sesquiterpenoid alcohols that generally account for greater than 90% of the total volatile release [27]. Variation in the abundance of certain VOCs, predominantly sesquiterpenes, has been noted between potato cultivars [28, 29]. Also, the presence of disease, insect herbivory and abiotic stress can alter volatile emissions [30]. Most prior related studies have focussed on the influence of VOCs on attraction of insect pests and/or virus vectors to potato foliage [29, 31, 32]. No similar studies on thrips interaction with potato volatiles have been done, but prior studies have indicated variation in thrips herbivory of potato foliage [7, 33], and studies with other plant hosts have documented the effects of plant volatiles on thrips feeding [34].

Here we attempt to confirm variation in *T*. *tabaci* preference amongst potato cultivars in a series of choice trials, and attempt to associate thrips non-preference with potato foliar volatiles. We used solid-phase microextraction (SPME) sampling to assess the relative abundance of volatiles captured within the headspace around the foliage of six potato cultivars that vary in their relative preference to *T*. *tabaci* (‘Atlantic’, ‘Russet Burbank’, ‘King Edward’, ‘Tasman’, ‘Bismark’), and two additional cultivars (‘Desiree’, Spunta’) at two different growth stages. We compared the volatile profiles of the sampled plants using Receiver Operating Characteristic Curve (ROC) analyses. The use of ROC curves is the most appropriate analysis tool for polytomous data where volatiles are used as predictors of biological traits, and has been commonly used for similar analyses in agricultural and medical research [35–38]. It can compare the usefulness of the predictors across all possible values of cutpoints for the predictors. This is a model-based approach, unlike more exploratory approaches, such as PCA, that may identify groupings but do not obtain predictors. We used this data to calculate the relative importance of individual volatiles in identifying thrips non-preference, plant age and specific cultivar. We can use this information to aid in breeding programs for insect resistance, and target specific compounds for further biological testing and subsequent pest management strategies.

## Materials and methods

### Potato plant material and thrips populations

Disease-free plants of eight potato cultivars; ‘Atlantic’, ‘Bismark’, ‘Desiree’, ‘King Edward’, ‘Russet Burbank’, ‘Shepody’, ‘Spunta’ and ‘Tasman’, were grown from mini-tubers produced from axenic tissue-cultures in a in a high health insect-proofed glasshouse (16–22°C) in plastic pots (20 cm in diameter, 20 cm in height, 4.7 L capacity) filled with potting mix containing sand, peat, and composted pine bark (10:10:80; pH 6.0) premixed with Osmocote 16–3.5–10 NPK resin-coated fertilizer (Scotts Australia Pty Ltd. Baulkham Hills, Australia).

Two colonies of thelytokous *T*. *tabaci* were used in host-preference trials. Both were collected from Cambridge, Tasmania, Australia (42°50’12” S, 147°26’28” E); population 1 collected from onion plants in 2002, and population 2 from potato plants in 2007. No specific permissions or permits were required for this study and the collection did not involve endangered or protected species. TSWV-free thrips colonies were reared from individual females on Common bean pods (*Phaseolus vulgaris* L.), following a protocol modified from van de Wetering et al. [39]. Colonies were reared in 7 cm x 9 cm containers, at 25°C ± 1°C, 65 ± 5% RH, with a 16-hour light and 8-hour dark photoperiod, using cool white fluorescent light under 450 *μ*mol-m^-2^s^-1^ photosynthetically active radiation (PAR), and maintained by transferral twice-weekly on to fresh bean pods.

### Thrips host-preference

The host-preferences for selected potato cultivars of *T*. *tabaci* were determined in a series of paired or multi-plant choice experiments (S1 Table).

#### Paired plant choice experiments

**Fig 1:**
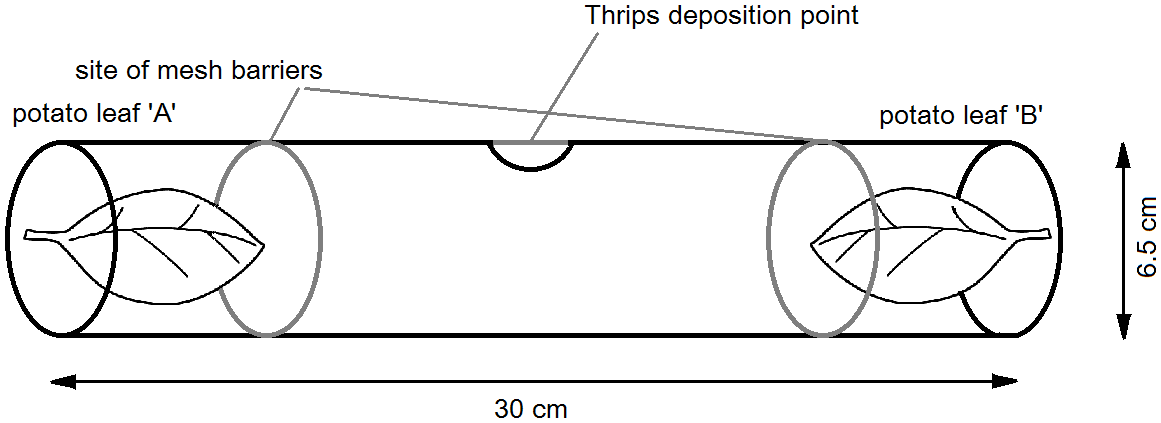
Schematic of experimental set-up for paired cultivars thrips preferences assays. Leaflets were detached from plants prior to use in apparatus. Adult onion thrips were deposited in the centre of the apparatus and thrips numbers colonising each cultivar leaflet assessed every 30 minutes for 3–4 hours after thrips introduction.

In these experiments detached leaflets of three potato cultivars (‘King Edward’, ‘Russet Burbank’, and ‘Bismark’) were tested against a leaflet of cv. ‘Bismark’ in paired choice assays. ‘Bismark’ was chosen as the standard comparator as prior field observations suggested probable thrips non-preference [7]. A terminal leaflet was detached from the youngest fully expanded leaf of seven week old plants of each potato cultivar and their petioles inserted into a 2 mL microfuge tube containing 0.7% sterile agarose. Leaves were then placed at either end of an experimental chamber consisting of a plastic tube (30 cm length, 6.5 cm diameter) with three 2.5 cm^2^ mesh covered vents along the upper chamber surface to prevent condensation build-up and the mixing of leaf volatiles. A small hole in the centre of the chamber allowed thrips to be introduced (Fig 1). Leaves were replaced with newly detached leaves from different plants of each cultivar between repeats. Approximately 25 Adult *T*. *tabaci* thrips (population 1) were collected from rearing containers, starved for 1 h and deposited in the centre of the chamber. Thrips migration to within 1 cm of the sample leaf at either end of the test chamber was recorded every 15–30 min for 3 h. Thrips failing to move to either of the chamber termini after 3 h were not recorded. The following paired challenges were conducted: ‘Bismark’ vs Blank (no leaflet); ‘Bismark’ vs ‘Bismark’; ‘Bismark’ vs ‘Russet Burbank’; ‘Bismark’ vs ‘King Edward’. Six replicates of each test pair were conducted with fresh cohorts of thrips, and cultivar leaflets rotated between each end of the chamber between replicates. The experiment was fully repeated except in this instance a fine mesh barrier was placed between leaf and the central part of the preference chamber at each end to remove potential visual cues. Both experiments took place at room temperature (*c*. 20°C) under indirect natural light at the same time of day.

#### Five plant choice experiments

**Fig 2:**
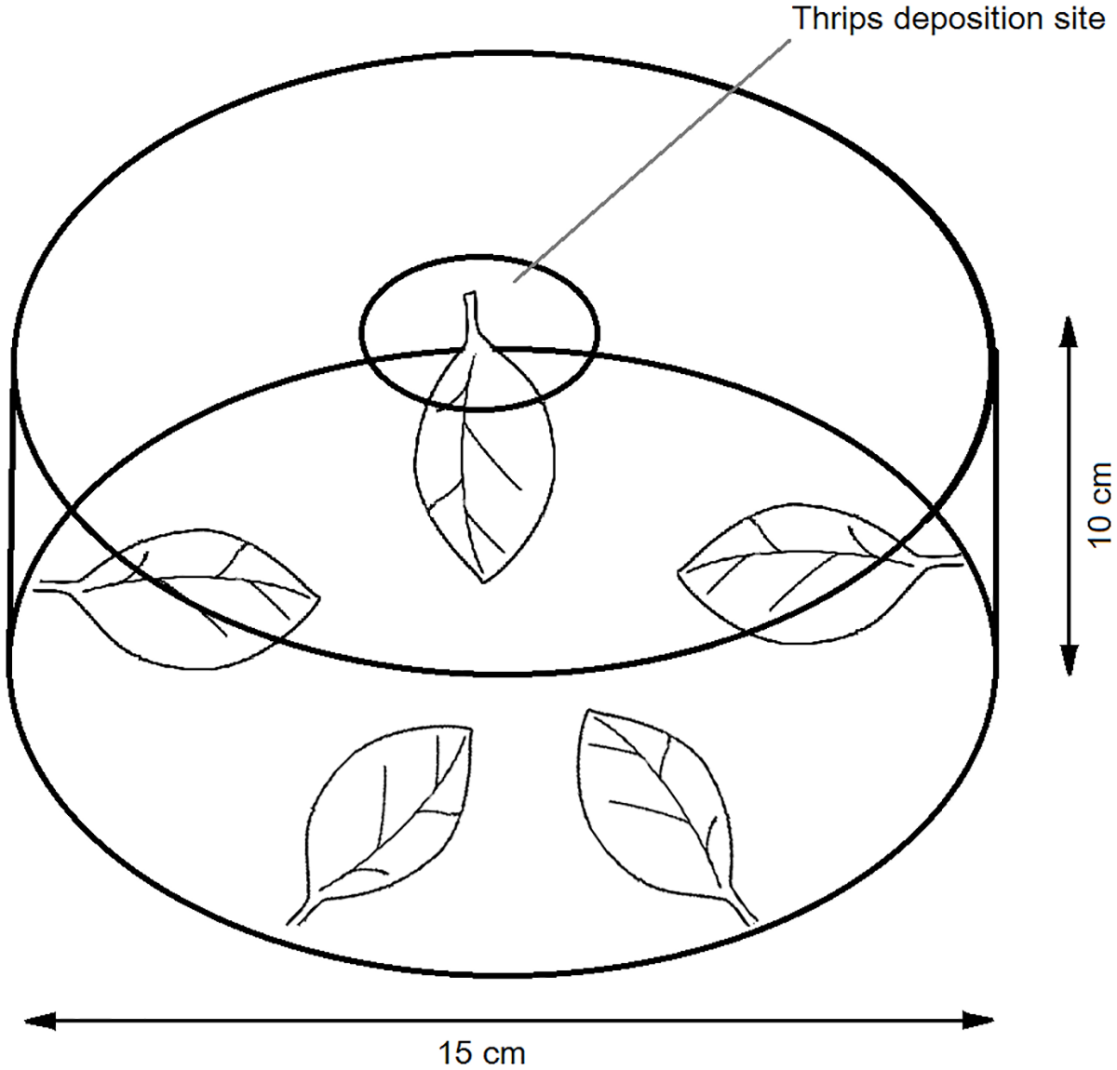
Schematic of experimental set-up for five cultivar thrips preferences assays. Leaflets remained attached to their parent plants. Adult onion thrips were deposited in the centre of the apparatus and thrips numbers colonising each cultivar leaflet assessed 180 minutes after thrips introduction.

In these experiments leaflets attached to plants of each of five cultivars (‘Atlantic’, ‘Bismark’, ‘Russet Burbank’, ‘Shepody’, and ‘Tasman’) were positioned within a circular plastic chamber (10 cm tall x 15 cm diameter; Fig 2). Planting material of ‘Kind Edward’ was not available for this experiment. Adult *T*. *tabaci* (*c*. 100 individuals from population 2) were placed in the centre of the chamber and the lid closed. After 3 h thrips present on the leaflet of each cultivar were carefully removed and counted. Thrips failing to migrate to a test leaflet were not recorded. This experiment was replicated six times with fresh cohorts of thrips, and was then fully repeated with a second set of plants.

### Volatile collection and analysis

Four plants each cultivar; one pair planted six weeks after the first pair; were grown in an insect-proofed glasshouse (16–22°C) for four (‘young’ plants) or ten (‘old’ plants) weeks post emergence prior to volatile analysis. At four weeks the plants were rapidly growing and had yet to initiate tuber set; at ten weeks plants had commenced tuber set but had yet to flower. The entire foliage of a single shoot from each potted potato plant was enclosed in a new polyethylene plastic bag (Clorox Pty Ltd., Padstow, Australia) which was loosely sealed around the potato stem. The bag was left in place for 30 minutes prior to volatile sampling. A manual SPME needle (75 μm Carboxen-PDMS, Supelco Inc., Bellefonte, PA, USA) was inserted through a puncture hole into the headspace of the plastic bag, and the needle exposed to the headspace volatiles for 15 minutes. A new bag was used for each plant.

Volatiles adsorbed onto the SPME needles were analysed by GC-MS using a Varian 3800 Gas Chromatograph coupled to a Varian 1200 quadrupole mass spectrometer operating in electron ionisation mode. A Varian 1177 injector was operated at 280°C in split mode, with a 2:1 split ratio. The column was a Varian FactorFour VF-5 ms, 30 m x 0.25 mm with 025 μm film. The carrier gas was helium at a constant flow of 1 mL/min. The column oven was held at 40°C for 3 minutes before a linear ramp to 240°C at 8°C per minute. The SPME syringe was left in the injector for ten minutes to minimise any carryover between samples. The transfer line was held at 290°C, the ion source at 220°C and electron voltage was 70 eV. The range from *m/z* 35 to 400 was scanned every 300 ms. Compounds were identified from a combination of mass spectral library matches (NIST library; NIST02, US Secretary of Commerce) and comprehensive in-house terpene libraries and relative retention times.

### Statistical analysis

Both the paired plant and five plant choice thrips preference trials were analysed as a series of challenges between cultivars. For the paired assays cv. ‘Bismark’ was always one of the alternatives. Both paired and the five-way data were analysed using logistic regression with a generalised logit link [40]. In the case of the paired comparisons the proportions of insects that chose Bismark over the alternative cultivar in each experiment and time over an alternative cultivar were compared. The five-way experiment data were analysed as a series of null models, cycling through each cultivar as reference level in turn. The results of the logistic regression were presented as log (base 10) odds, which represented the (log) odds of selecting the first cultivar over the alternative. All analyses were conducted using Proc Logistic in SAS version 9.3.

For volatile analysis, Receiver Operating Characteristic (ROC) curves were used to characterise the usefulness of each volatile as a predictor of each trait of interest. In each ROC curve the estimated true positive (tp) rate was plotted on the vertical axis while the estimated false positive (fp) rate was plotted on the horizontal axis. The estimated tp rate was the proportion of samples with the predictor value being above a particular cut-point given that the sample is in the target population. The estimated fp rate was the proportion of samples above the same cut-point where the sample is not in the target population. ROC’s were constructed using the ROCR package in R [41]. Each ROC plot examines the relationship of the tp’s and fp’s. Ideally, where a predictor is a useful one, the curve rises rapidly to the top-left corner or the bottom right corner and then flattens to follow the top or bottom of the plot. In the case where a predictor is not informative, the curve follows the diagonal line from the bottom left to the top-right. The areas under the ROC curve (AUROC) were calculated to provide an indication of the test performance regardless of the selected cut-point. The AUROC can range from 0 to 1.0; those tests with areas closer to 0.5 have little discriminatory power whereas those tests with areas closer to 0 or 1 are good discriminators. The AUROC can also be interpreted as the average probability of correctly predicting a positive case across all possible cut-points of the predictor. To interpret the diagnostic accuracy of the tests, we consider AUROCs between 0.3 and 0.5 or 0.5 and 0.7 to reflect low accuracy; those between 0.2 and 0.3 or 0.7 and 0.8 have moderate accuracy; 0.1 and 0.2 or 0.8 and 0.9 show high accuracy; and those between 0.0 and 0.1 or 0.9 and 1.0, very high accuracy [42].

## Results

### Thrips host preference

**Fig 3:**
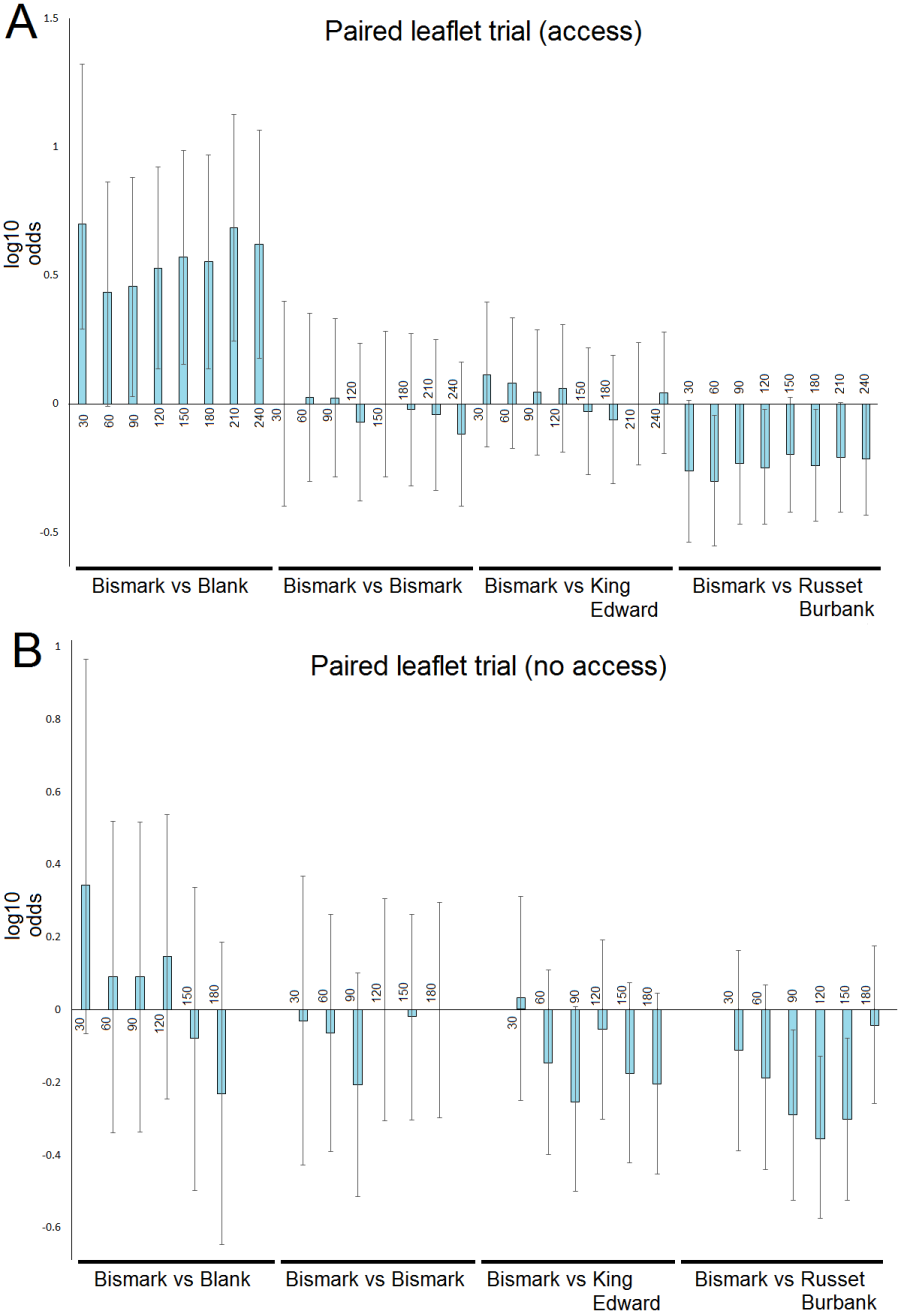
Onion thrips preference in paired potato leaflet assays. In trial 1 (A) leaflets were available for thrips, the trial 2 (B) leaflet were screened from thrips by a white mesh presenting visual stimuli and leaflet contact. The X axis indicates time (min) from deposition of thrips within the apparatus, the Y axis is the log value of the odds ratio; a positive value indicates preference for ‘Bismark’ leaflet, a negative value indicate preference for alternate cultivar or treatment. Error bars indicate the 95% confidence limits. Where the confidence limits do not encompass Y = 0 then the preference is significant (P<0.05).

Significant differences in thrips preference were noted in the two paired plant choice experiments that compared cultivar treatments against a leaflet of ‘Bismark’. In trial 1, where thrips had access to the test leaflets, the thrips showed a marked preference for the ‘Bismark’ leaflet over the blank treatment that was statistically significant at the 30 (P = 0.041), 90 (P = 0.05), 120 (P = 0.011), 150 (P = 0.009), 180 (P = 0.012) and 240 min (P = 0.002) measurements, and a preference for the ‘Russet Burbank’ leaflet over the ‘Bismark’ leaflet that was statistically significant at the 60 (P = 0.026), 120 (P = 0.034) and 180 min (P = 0.041) assessments. In contrast there was no differences in preference shown between the paired ‘Bismark’ leaflets, and between the ‘Bismark’ leaflet and the ‘King Edward’ leaflet (Fig 3).

In trial 2, where thrips were screened from access to the leaflets by mesh, similar results were seen except for the ‘Bismark’ leaflet and blank test, where no obvious trends were observed. The preference for ‘Russet Burbank’ was again only statistically significant at three (90–150 min) of the measurement periods (Fig 3).

**Fig 4:**
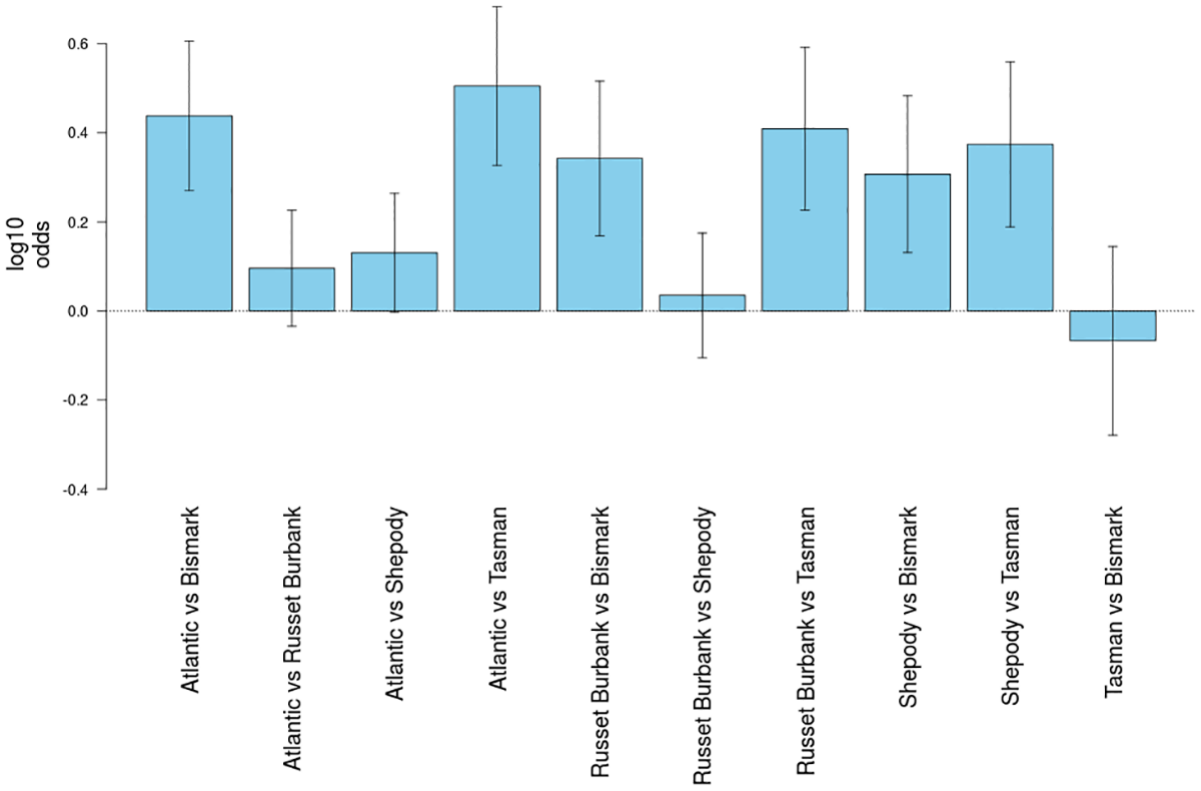
Onion thrips preference in five potato cultivar preference tests. Data from two preference trials were combined for analysis. The X axis indicates the cultivar pairing under interrogation, the Y axis is the log value of the odds ratio, a positive value indicates preference for the first named cultivar, a negative value indicate preference for second named cultivar. Error bars indicate the 95% confidence limits. Where the confidence limits do not encompass Y = 0 then the preference is significant (P<0.05).

Differences in host preference choice by *T*. *tabaci* was again found in both five plant choice experiments. Insect recovery numbers following release varied from 75 to 16 individuals. The mean proportion of thrips recorded on each cultivar were: ‘Atlantic’ (26% in trial 1, 19% in trial 2); ‘Russet Burbank’ (17%, 21%); ‘Shepody’ (19%, 13%); ‘Tasman’ (15%; 0%); and ‘Bismark’ (11%, 6%). For greater analytical power, and to deal with the zero value recorded for ‘Tasman’ in trial 2, the data for both trials were combined for analysis and generation of log odds ratios (Fig 4). Significant preferences was shown for ‘Atlantic’ (P<0.0001; P<0.0001), ‘Shepody’ (P<0.0001; P = 0.0006) and ‘Russet Burbank’ (P<0.0001; P = 0.0001) when compared with ‘Tasman’ or ‘Bismark’. No differences in thrips preference were seen in comparisons between ‘Atlantic’ and ‘Russet Burbank’; ‘Russet Burbank’ and ‘Shepody’, and ‘Tasman’ and ‘Bismark’. A minor but significant preference for ‘Atlantic’ over ‘Shepody’ was also shown (Fig 4).

### Volatile analysis

Thirty two distinct volatiles were identified from headspace sampling of potato foliage using Carboxen-PDMS SPME needles. These were two aliphatic aldehydes, an aliphatic alcohol, an aromatic alcohol, four monoterpenes, and 24 sesquiterpenes. The sesquiterpene region was extremely complex and not all compounds were unequivocally identified, so several were assigned as unknown sesquiterpenes A to K. (Table 1). Caryophyllene was the most intense signal detected in the SPME chromatogram, being between 33–61% of the total ion current (TIC) detected in each sample (Table 1). All other compounds were less than 14% of the TIC. It should be stressed that these figures do not represent the actual proportion of these compounds either in the headspace or in the leaf, but simply the proportion they represented of the Total Ion Current. A compound’s vapour pressure and specific affinity for the SPME liquid phase strongly affect its contribution to the TIC [43]. Not all compounds were detected in each cultivar. Undetected compounds were myrcene from ‘Atlantic’, tricylene from ‘Tasman’, α-santalene from ‘Bismark’, and (*Z*)-β-farnesene from ‘King Edward’ and ‘Spunta’ samples (Table 1).

**Table 1:** The mean proportion of 32 predominately terpene volatiles detected within the sampled head space of eight potato varieties at two plant ages.

Run time (min)^a^	Volatile^b^	Potato variety															
		*T*. *tabaci* preference^c^						*T*. *tabaci* non-preference						Not tested			
		Atlantic		Russet Burbank		Shepody		King Edward		Tasman		Bismark		Spunta		Desiree	
		Y^d^	O	Y	O	Y	O	Y	O	Y	O	Y	O	Y	O	Y	O
4.048	isoamyl alcohol	0.002	0.005	0.005	0.017	0.004	0.005	0.019	0.025	0.009	0.003	0.018	0.017	0.002	0.003	0.024	0.002
5.402	hexanal	0.001	0.016	0.002	0.003	0.002	0.014	0.000	0.001	0.002	0.000	0.006	0.021	0.001	0.017	0.013	0.013
6.711	(*E*)-2-hexenal	0.004	0.026	0.001	0.003	0.012	0.005	0.000	0.002	0.008	0.000	0.017	0.018	0.005	0.099	0.035	0.021
8.223	tricyclene	0.004	0.001	0.001	0.001	0.001	0.002	0.006	0.003	0.000	0.000	0.000	0.001	0.007	0.008	0.009	0.015
8.475	α-thujene	0.006	0.001	0.001	0.001	0.002	0.002	0.007	0.003	0.001	0.001	0.004	0.005	0.003	0.001	0.008	0.006
9.708	myrcene	0.000	0.000	0.009	0.014	0.024	0.040	0.015	0.011	0.008	0.010	0.009	0.013	0.007	0.010	0.017	0.016
10.626	limonene	0.001	0.001	0.002	0.002	0.004	0.001	0.001	0.015	0.001	0.002	0.004	0.006	0.004	0.003	0.010	0.011
12.395	phenylethanol	0.001	0.002	0.000	0.002	0.006	0.007	0.045	0.030	0.002	0.002	0.004	0.000	0.001	0.001	0.002	0.001
16.423	sesqui A	0.005	0.004	0.006	0.008	0.006	0.003	0.012	0.002	0.003	0.002	0.010	0.011	0.007	0.004	0.015	0.004
16.673	sesqui B	0.005	0.004	0.014	0.016	0.013	0.014	0.011	0.007	0.009	0.007	0.011	0.010	0.009	0.008	0.010	0.007
17.188	sesqui C	0.003	0.003	0.008	0.009	0.009	0.007	0.004	0.006	0.006	0.005	0.010	0.008	0.002	0.005	0.004	0.004
17.310	sesqui D	0.002	0.004	0.001	0.000	0.004	0.005	0.001	0.001	0.001	0.002	0.005	0.002	0.002	0.005	0.003	0.007
17.381	β-elemene	0.035	0.032	0.049	0.048	0.050	0.035	0.073	0.078	0.054	0.043	0.071	0.070	0.039	0.024	0.037	0.029
17.698	α-gurjunene	0.003	0.009	0.008	0.006	0.005	0.006	0.015	0.018	0.006	0.006	0.008	0.015	0.006	0.008	0.011	0.010
17.861	α-santalene	0.002	0.003	0.001	0.002	0.003	0.006	0.003	0.002	0.001	0.002	0.000	0.000	0.002	0.004	0.003	0.007
17.955	caryophyllene	0.507	0.485	0.554	0.452	0.332	0.343	0.499	0.550	0.482	0.479	0.454	0.480	0.613	0.483	0.430	0.472
18.075	(*E*)-α-bergamotene	0.022	0.032	0.015	0.023	0.026	0.042	0.010	0.008	0.015	0.025	0.005	0.004	0.024	0.033	0.019	0.036
18.183	sesqui E	0.004	0.006	0.001	0.002	0.005	0.008	0.001	0.001	0.002	0.003	0.002	0.000	0.002	0.006	0.003	0.008
18.254	sesqui F	0.017	0.021	0.014	0.017	0.019	0.023	0.012	0.013	0.017	0.020	0.014	0.019	0.018	0.019	0.027	0.036
18.306	(*Z*)-β-farnesene	0.057	0.051	0.060	0.080	0.057	0.068	0.000	0.000	0.068	0.078	0.135	0.134	0.000	0.000	0.019	0.019
18.537	humulene	0.069	0.070	0.029	0.025	0.071	0.061	0.071	0.076	0.075	0.073	0.031	0.029	0.031	0.025	0.027	0.027
18.625	sesqui G	0.011	0.010	0.023	0.027	0.031	0.021	0.018	0.018	0.017	0.013	0.023	0.022	0.023	0.017	0.019	0.019
18.818	α-curcumene	0.023	0.024	0.013	0.019	0.021	0.023	0.015	0.009	0.019	0.021	0.007	0.006	0.014	0.022	0.018	0.027
18.920	(*E*)-β-farnesene	0.056	0.050	0.036	0.055	0.076	0.091	0.028	0.015	0.029	0.041	0.035	0.011	0.052	0.065	0.113	0.081
19.017	zingiberene	0.010	0.011	0.004	0.007	0.015	0.012	0.005	0.001	0.011	0.013	0.001	0.000	0.006	0.010	0.005	0.012
19.160	sesqui H	0.016	0.014	0.030	0.022	0.023	0.014	0.017	0.017	0.019	0.013	0.031	0.027	0.017	0.011	0.011	0.012
19.232	sesqui I	0.017	0.020	0.007	0.011	0.011	0.013	0.002	0.002	0.020	0.024	0.010	0.007	0.005	0.009	0.010	0.010
19.394	sesqui J	0.019	0.012	0.021	0.022	0.036	0.021	0.033	0.027	0.027	0.017	0.021	0.013	0.012	0.011	0.013	0.007
19.451	δ-cadinene	0.009	0.010	0.020	0.022	0.020	0.017	0.020	0.010	0.017	0.016	0.011	0.009	0.014	0.010	0.008	0.010
19.486	β-sesquiphellandrene	0.056	0.050	0.008	0.025	0.049	0.042	0.008	0.005	0.032	0.046	0.001	0.001	0.031	0.044	0.037	0.035
19.522	calamenene	0.026	0.019	0.048	0.049	0.049	0.041	0.036	0.033	0.030	0.026	0.039	0.037	0.037	0.031	0.032	0.032
19.751	sesqui K	0.007	0.004	0.009	0.009	0.013	0.008	0.012	0.011	0.009	0.006	0.005	0.004	0.007	0.004	0.007	0.005

^a^ Retention time in column (minutes)

^b^ sesqui A-K are unidentified sesquiterpenes

^c^ cultivars showed preference or non-preference to *Thrips tabaci* in paired or 5-way choice experiments or where not tested.

^d^ Y = young plant (4 weeks); O = old plant (10 weeks).

**Fig 5:**
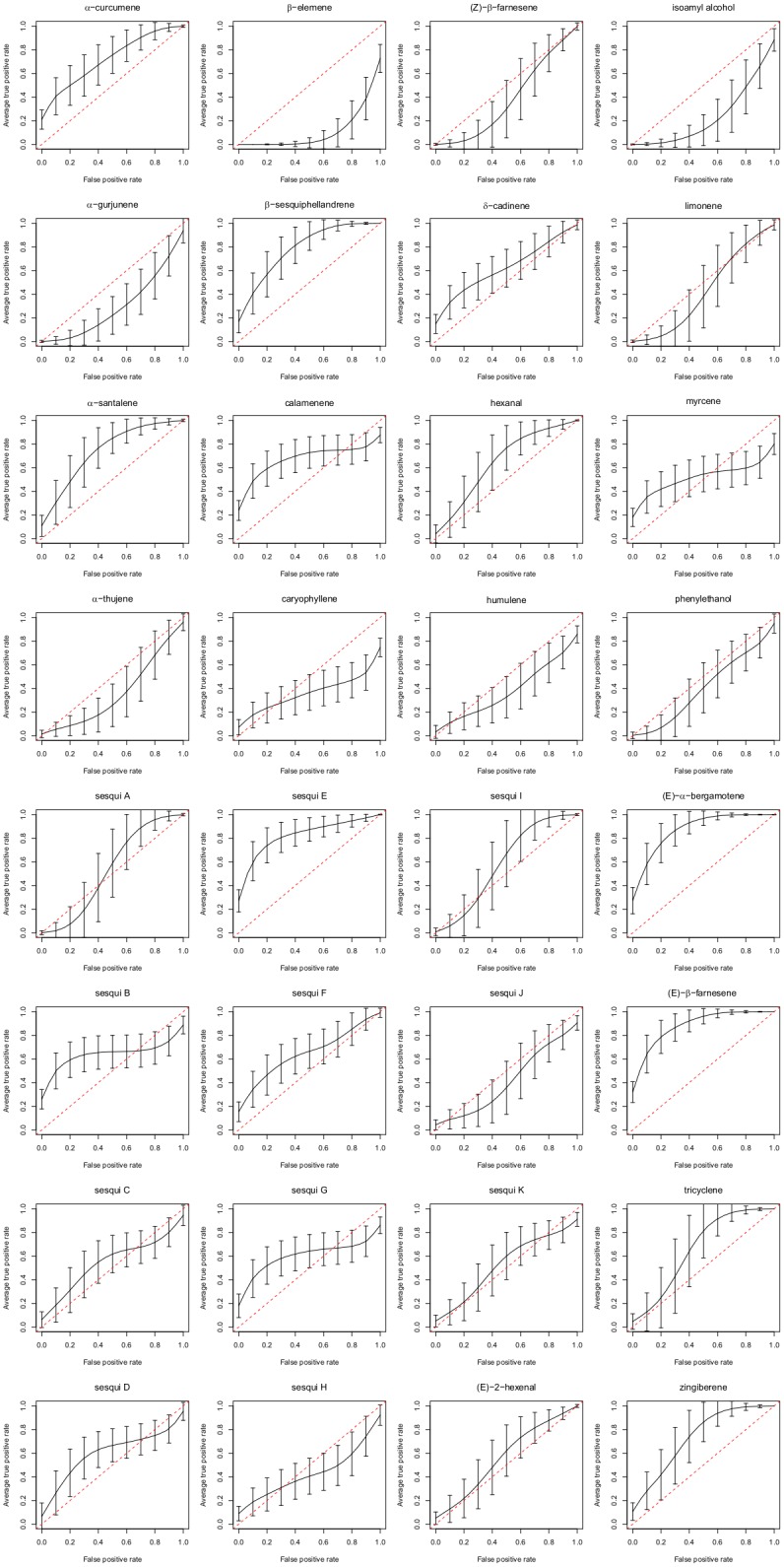
Receiver Operating Characteristic curves for each potato leaf volatile for the thrips preference trait. Curves significantly above the diagonal line indicate thrips attraction, curves significantly below the diagonal line indicate thrips deterrence. Error bars indicate the 95% confidence limits.

Examination of the proportion of each volatile by ROC curve analysis identified volatiles that were important predictors of each cultivar, and the thrips preference and plant age traits (Table 2; Fig 5). For thrips non-preference, β-elemene (0.09 area under curve; AUROC), isoamyl alcohol (0.22) and α-gurjunene (0.30) showed a significant positive association and may therefore be associated with thrips repulsion. *(E*)-β-farnesene (0.90), *(E*)-α-bergamotene (0.89), sesqui E (0.84), β-sesquiphellandrene (0.80), α-santalene (0.76), zingiberene (0.75) and α-curcumene (0.74) all showed significant lack of association with the non-preference trait and may therefore be associated with thrips attraction to potato foliage (Table 2; Fig 5). For plant age, hexanal (0.74), *(E*)-α-bergamotene (0.72) and sesqui F (0.71) were significantly associated with the older plants and sesqui A (0.25), sesqui H (0.29) and sesqui J (0.30) with the younger plants (Table 2). Volatiles significantly associated with individual cultivars present in greater or lesser relative amounts than other cultivars tested were also identified, with AUROC values as great as 1.00 or as little as 0.00 (Table 2).

**Table 2:** Calculated area under Receiver Operator Characteristic curves (AUROC) for each volatile against each measured trait^a^.

Volatiles^b^	Onion thrips non-preference^c^	Plant age^d^	Potato variety^e^							
			Atlantic	Russet Burbank	Shepody	Bismark	King Edward	Tasman	Spunta	Desiree
α-curcumene	0.74	0.60	0.28	0.41	0.67	0.95	0.72	0.63	0.52	0.23
α-gurjunene	0.29	0.64	0.66	0.38	0.25	0.29	0.25	0.30	0.54	0.18
α-santalene	0.76	0.68	0.38	0.29	0.79	0.96	0.49	0.36	0.44	0.17
α-thujene	0.36	0.45	0.65	0.19	0.56	0.35	0.31	0.24	0.56	0.12
β-elemene	0.09	0.34	0.81	0.59	0.43	0.10	0.04	0.58	0.83	0.82
β-sesquiphellandrene	0.80	0.60	0.13	0.27	0.75	0.97	0.85	0.66	0.34	0.39
calamenene	0.68	0.40	0.88	0.92	0.85	0.37	0.54	0.22	0.56	0.63
caryophyllene	0.35	0.46	0.47	0.64	0.00	0.56	0.25	0.53	0.22	0.67
(*Z*)-β-farnesene	0.41	0.54	0.47	0.68	0.60	0.00	0.93	0.75	0.93	0.72
δ-cadinene	0.63	0.39	0.79	0.94	0.75	0.77	0.45	0.70	0.60	0.80
hexanal	0.67	0.75	0.47	0.44	0.61	0.32	0.84	0.26	0.45	0.20
humulene	0.37	0.42	0.22	0.11	0.69	0.66	0.17	0.84	0.74	0.86
isoamyl alcohol	0.22	0.48	0.74	0.62	0.39	0.16	0.10	0.34	0.82	0.52
limonene	0.43	0.50	0.75	0.43	0.39	0.32	0.56	0.24	0.33	0.08
myrcene	0.52	0.58	1.00	0.53	1.00	0.56	0.37	0.30	0.72	0.22
phenylethanol	0.40	0.53	0.61	0.31	0.85	0.60	0.00	0.49	0.77	0.65
sesqui A	0.54	0.24	0.56	0.75	0.37	0.08	0.67	0.14	0.50	0.44
sesqui B	0.65	0.40	0.98	0.96	0.86	0.37	0.52	0.35	0.65	0.64
sesqui C	0.55	0.51	0.90	0.87	0.79	0.10	0.63	0.46	0.74	0.75
sesqui D	0.61	0.67	0.45	0.21	0.76	0.39	0.75	0.30	0.44	0.25
sesqui E	0.85	0.69	0.27	0.32	0.85	0.79	0.81	0.39	0.40	0.29
sesqui F	0.66	0.71	0.42	0.30	0.70	0.62	0.85	0.56	0.46	0.23
sesqui G	0.61	0.39	0.99	0.89	0.82	0.25	0.62	0.20	0.49	0.57
sesqui H	0.43	0.27	0.64	0.75	0.56	0.09	0.43	0.43	0.65	0.91
sesqui I	0.59	0.60	0.14	0.39	0.62	0.60	0.97	0.97	0.73	0.54
sesqui J	0.41	0.30	0.66	0.62	0.76	0.59	0.09	0.66	0.83	0.87
sesqui K	0.52	0.33	0.70	0.73	0.71	0.78	0.10	0.54	0.75	0.63
(*E*)-2-hexenal	0.58	0.66	0.40	0.28	0.56	0.25	0.85	0.29	0.35	0.30
(*E*)-α-bergamotene	0.89	0.72	0.29	0.38	0.86	0.96	0.87	0.47	0.22	0.35
(*E*)-β-farnesene	0.91	0.53	0.46	0.44	0.92	0.83	0.90	0.28	0.35	0.11
tricyclene	0.68	0.53	0.47	0.27	0.52	0.88	0.30	0.04	0.15	0.02
zingiberene	0.74	0.58	0.38	0.32	0.94	0.95	0.82	0.82	0.48	0.44

^a^ Data are measures of how important each volatile is as a predictor of each trait over all possible values of the cut point under the receiver operator curve. Values approaching 0 or 1 indicate high importance in predicting the trait in a positive or negative manner, values approaching 0.5 indicate random chance.

^b^ sesqui A-K are unidentified sesquiterpenes

^c^ Thrips non-preference determined experimentally for subsets of the tested varieties using *Thrips tabaci*. A low score is indicative of non-preference, a high score is indicative of preference.

^d^ Comparison of plant age across varieties. A low score is indicative of young plants (4 weeks old), a high score is indicative of old plants 10 weeks old).

^e^ Data indicates relative importance of each volatile in distinguishing each variety from the others. A low score is indicative of the variety, a high score is indicative of the other varieties.

## Discussion

In the present study we show that potato cultivars ‘Bismark’, ‘Tasman’ and ‘King Edward’ are less preferred by *T*. *tabaci* than ‘Atlantic’, ‘Russet Burbank’ and ‘Shepody’. Previous studies have shown that thrips may exhibit differences in host preference between individual cultivars of potato [7, 33, 44], and other crop plants including lettuce (*Lactuca sativa* L.) [45], capsicum (*Capsicum annuum* L.) [46, 47], onion (*Allium cepa* L.) [48, 49], cucumber (*Cucumis sativus* L.) [50], *Chrysanthemum* [51], *Impatiens* [52] and rose (*Rosa* spp.) [53]. The immediate parentage of the three non-preferred cultivars does not suggest a common heritage. ‘King Edward’ was bred in the United Kingdom in 1902 from ‘Magnum Bonham’ x ‘Beauty of Hebron’ parents, ‘Tasman’ was bred in Australia in 1974 from (‘Duke of York’ x BC 0/4) x (‘Crana’ x 11–79) parents, and whilst the parentage of ‘Bismark’ is unknown it is believed to be derived from the ‘Bismarck’ (bred in Germany in 1889 from parents ‘Sachsiche zwiebel’ x ‘Erste van Fromsdorf’) [54].

Testing for insect preference in plants can be difficult and is prone to experimental bias with set-up and environmental factors influencing outcomes. As tests were carried out with multiple thrips per area in both paired and five plant choice tests we acknowledge that the thrips may have responded to one another through visual, volatile or tactile cues that may have influenced the outcome. Similarly in the five plant choice test the volatiles from each cultivar could mix to some extent and influence thrips behaviour. Importantly, the cultivars associated with the thrips non-preference phenotype were observed using two distinct experimental systems (pair-wise and five plant choice testing), with both intact and detached leaves providing the volatile cues, and reflect prior field observations (for ‘Bismark’). The use of multiple systems can reduce the influence of inherent experimental bias that may be present in single systems. We also showed restriction of visual cues did not alter non-preference of cv. ‘Bismark’. These observations together suggest the observed thrips preference phenotypes are robust. We also note that the study reported here only tested two thrips populations. In Australia all recorded *T*. *tabaci* populations are thelytokous reproducing parthenogenetically limiting genetic plasticity [55]. A prior study of the genetic diversity of Australian *T*. *tabaci* populations revealed two distinct biotypes segregating by collection host and capacity to vector TSWV [55]. In the present study the two populations were sourced from potato hosts associated with the TSWV vector capable biotype and thus are likely to represent field populations of interest to TSWV epidemics in potato.

Analysis of the headspace of foliage from eight potato cultivars assessed in this study using Carboxen-PDMS SPME needles and GC-MS revealed 32 volatiles including two aliphatic aldehydes, an aliphatic alcohol, an aromatic alcohol, four monoterpenes, and 25 sesquiterpenes, the latter including 11 unidentified compounds. Isoamyl alcohol, α-thujene, α-santalene, α-curcumene and calamene appear to be previously unreported VOCs from potato foliage [27–29, 31, 56, 57]. We note SPME allows accurate comparison of specific compounds across samples, whereas comparison of amounts of different compounds within samples is not possible by relative SPME quantification [43]. For this reason we expressed compounds as a percentage of the total signal and then comparison of that percentage between samples (e.g. cultivars) is quite valid. In all samples Caryophyllene gave the most intense signal. Prior studies using a range of different sampling and analysis technologies have shown caryophyllene to be the most abundant VOC in the headspace surrounding, and on the surface of potato foliage [27, 29], although in the variety Chipeta, γ-elemene was detected in greater abundance [29]. It is important to note that different headspace sampling methods can result in altered ratios of volatiles depending on sampling conditions [58] and abundance results may not necessarily be directly compared or indeed appropriate (as for SPME). It is also known that volatile release by potato plants can be affected by the time of day with greatest releases occurring in the afternoon this being more evident in older plants [59]. In our study we ensured that all comparable insect preference trials and volatile samplings occurred at the same time during the day.

Examination of the proportion of potato VOCs by ROC curve analysis showed that onion thrips preference traits, plant age and cultivar identity had volatiles that were important predictors of these factors. Previous studies have shown the role of individual or VOC cocktails from potato in altering behaviour response of insects. Analysis of antennal activity in Guatemalan moth females of individual potato volatiles showed significant activity with several volatiles of which β-myrcene, β-carophyllene, (*E*)-β-farnesene (*T*. *tabaci* preference), and δ-cadinene were also identified in our study [27]. There are also specific effects of VOC blends from potato that are known to affect aphid choice and it may be the relative concentration of chemicals within a mixture that serve as cues [60]. Indeed it has been shown that VOCs responsible for host-cues in a blend can become non-host cues when presented individually [61]. Certain plant volatiles may also mask attractive compounds when present in abundance. For example, (*E*)-2-hexen-1-ol and (*E*)-2-hexenal, whilst showing no repellent activity themselves, reduced the attraction of Colorado potato beetle to potato odours [62]. Thus interpretation of our results on thrips preference requires recognition that important VOCs identified may require other compounds for activity. Of the compounds identified with significant association to *T*. *tabaci* repellency, β-elemene has been identified as a major constituent in the essential oil from *Cinnamomum myrrha* that was repellent to Legume flower thrips [63], and isoamyl alcohol is known to attract hornets [64]. Similarly, of those compounds that were associated with *T*. *tabaci* attraction, *(E*)-β-farnesene is well known as a repellent of aphids [65–67], but has been previously shown to attract western flower thrips [68]. Zingiberene in a cocktail mix has been shown to attract the two-spotted stink bug [69].

Distinct foliar volatile patterns were found for each of the cultivars tested. Prior studies, using different extraction and detection methodologies, also found distinct foliar VOC patterns for different potato cultivars [28, 29]. Using direct extraction in methylene chloride and GC, GC-MS and NMR analyses, Szafranek et al. [28] identified two sesquiterpenoid alcohols and 17 sesquiterpenes, eight of which were in common with the present study, although further characterisation of the unidentified compounds from both studies may reveal further commonalities. They also found that VOC patterns could be used to differentiate cultivars. Similarly, an analysis of potato volatiles from late-blight affected plants revealed specific volatiles that were indicators of infection; these being (*E*)-2-hexenal, 5-ethyl-2(5H)-furanone and 2-phenylethanol [30].

Few studies have examined changes in VOC patterns with potato plant age. Karlsson et al. [27] examined VOC profiles from potato foliage at sprouting, tuberisation and flowering, and compared these with volatiles produced from tubers. They showed that while most compounds detected were common at all three phenological stages, the release rate of many sesquiterpenes increased between sprouting and flowering. Flowering-related volatiles were also identified. They did not however attempt to identify the relative importance of each volatile in prediction of plant development.

In conclusion, we have demonstrated potato cultivars differ in their attractiveness to *T*. *tabaci*, and that analysis of headspace foliar volatiles reveals individual VOCs statistically associated with thrips preference and non-preference. Furthermore, foliar volatiles were associated with plant age and with individual cultivar. These data and techniques can be applied to potato breeding to assist in selection of thrips non-preference to aid in TSWV control, and for identification of cultivars. Furthermore, VOCs associated with thrips preference traits may be now examined as potential tools for pest and virus management as thrips lures or in a push:pull system [25, 70, 71].

## Supporting information

S1 Table Experimental details for the thrips preference trials. (DOCX)

## References

[1] Wilson CR. Applied Plant Virology. Wallingford, UK: CABI Press; 2014.

[2] de Bokx JA, van der Want JPH. Viruses of potato and seed potato production. Wageningen, the Netherlands: Purdoc; 1987.

[3] Abad JA, Moyer JW, Kennedy GG, Holmes GA, Cubeta MA. Tomato spotted wilt virus on Potato in Eastern North Carolina. Am J Potato Res. 2005; 82: 255–261.

[4] Norris DO. Spotted wilt of potato. I The field disease and studies of the causal agent. Aust J Agric Res. 1951; 2: 221–242.

[5] Wilson CR. Resistance to infection and translocation of tomato spotted wilt virus in potatoes. Plant Pathol. 2001; 50: 402–410.

[6] Latham IA, Jones RAC. Occurrence of tomato spotted wilt tospovirus in native flora, weeds and horticultural crops. Aust J Agric Res. 1997; 48: 359–369.

[7] Jericho C. Jnr. Epidemiology and the development of risk assessment models for the management of tomato spotted wilt virus (TSWV) in potatoes. Ph.D. Thesis, University of Tasmania. 2005.

[8] Beck SD. Resistance of plants to insects. Ann Rev Entomol. 1965; 10: 207–232.

[9] Berlandier FA, Thackray DJ, Jones RAC, Latham LJ, Cartwright L. Determining the relative roles of different aphid species as vectors of cucumber mosaic and bean yellow mosaic viruses in lupins. Ann. Appl. Biol. 1997; 131: 297–314.

[10] Wilson CR, Jones RAC. Virus content of seed potato stocks produced in a unique seed production scheme. Ann Appl Biol. 1990; 116: 103–109.

[11] Fraser RSS. The genetics of resistance to plant viruses. Ann Rev Phytopathol 1990; 28: 179–200.

[12] Wilson CR, Jones RAC. Resistance to potato leafroll virus infection and accumulation in potato cultivars and the effects of previous infection with other viruses on expression of resistance. Aust J Agric Res. 1993; 44: 1891–1904.

[13] Roggero P, Masenga V, Tavella L. Field isolates of *Tomato spotted wilt virus* overcoming resistance in pepper and their spread to other hosts in Italy. Plant Dis. 2002; 86: 950–954. 10.1094/PDIS.2002.86.9.950 30818554

[14] Aramburu J, Marti M. The occurrence in north-east Spain of a variant of *Tomato spotted wilt virus* (TSWV) that breaks resistance in tomato (*Lycopersicon esculentum*) containing the *Sw-5* gene. Plant Pathol. 2003; 52: 407.

[15] Painter RH. Insect resistance in crop plants. New York: Macmillan 1951.

[16] Jones AT. Control of virus infection in crop plants through vector resistance: a review of achievements, prospects and problems. Ann Appl Biol. 1987; 111: 745–772.

[17] Paré PW, Tumlinson JH. Plant volatiles as a defense against insect herbivores. Plant Physiol. 1999; 121: 325–332. 10517823 PMC1539229

[18] Metcalf RL, Metcalf ER. Plant kairomones in insect ecology and control. Chapman and Hall Ltd; 1992.

[19] Visser JH. Host odor perception in phytophagous insects. Ann Rev Entomol. 1986; 31: 121–44.

[20] Foster SP, Harris MO. Behavioral manipulation methods for insect pest-management. Annual Rev Entomol. 1997; 42: 123–146. 15012310 10.1146/annurev.ento.42.1.123

[21] Koschier EH. Plant allelochemicals in thrips control strategies. Adv Phytomed. 2006; 3: 221–249.

[22] Curtis CE, Clark JD. Responses of navel orangeworm moths to attractants evaluated as oviposition stimulants in an almond orchard. Environ Entomol. 1979; 8: 330–333.

[23] Hern A, Dorn S. A female-specific attractant for the codling moth, Cydia pomonella from apple fruit volatiles. Naturwissenschaften. 2004; 91: 77–80. doi: 10.1007/s00114-003-0484-6 14991144 10.1007/s00114-003-0484-6

[24] Nottingham SF, Hardie J, Dawson GW, Hick AJ, Pickett JA, Wadhams LJ, Woodcock CM. Behavioral and electrophysiological responses of aphids to host and nonhost plant volatiles. J Chem Ecol. 1991; 17: 1231–1242. doi: 10.1007/BF01402946 24259180 10.1007/BF01402946

[25] Pickett JA, Bruce TJ, Chamberlain KE, Hassanali AH, Khan ZR, Matthes MC, et al Plant volatiles yielding new ways to exploit plant defence In: Dicke M, Takken W, editors. Chemical Ecology: from Gene to Ecosystem. Springer 2006 pp. 161–73.

[26] Szendrei Z, Roderigues-Saona C. 2010 A meta-analysis of insect behavioural manipulation with plant volatiles. Entomol Exp Appl. 2010; 134: 201–210.

[27] Karlsson MF. Birgersson G, Prado AMC, Bosa F, Bengtsson M, Witzgall P. Plant odor analysis of potato: response of Guatemalan moth to above-and belowground potato volatiles. J Agric Food Chem. 2009; 57: 5903–5909. doi: 10.1021/jf803730h 19496533 10.1021/jf803730h

[28] Szafranek B, Chapkowska K, Pawinska M, Szafranek J. Analysis of leaf surface sesquiterpenes in potato varieties. J Agric Food Chem. 2005; 53: 2817–2822. doi: 10.1021/jf040437g 15826024 10.1021/jf040437g

[29] Rajabaskar D, Ding H, Wu Y, Eigenbrode SD. Behavioural responses of Green Peach aphid, *Myzus persicae* (Sulzer), to the volatile organic compound emissions from four potato varieties. Am J Potato Res. 2013; 90: 171–178.

[30] Laothawornkitkul J, Jansen RMC, Smid HM, Bouwmeester HJ, Muller J, van Bruggen AHC. Volatile organic compounds as a diagnostic marker of late blight infected potato plants: A pilot study. Crop Prot. 2010; 29: 872–878.

[31] Eigenbrode SD, Ding H, Shiel P, Berger PH. Volatiles from potato plants infected with potato leafroll virus attract and arrest the virus vector *Myzus persicae* (Homoptera: Aphidae). Proc R Soc Lond [Biol]. 2002; 269: 455–460. 10.1098/rspb.2001.1909 PMC1690919 11886636

[32] Werner BJ, Mowry TM, Bosque-Perez NA, Ding H, Eigenbrode SD. Changes in Green Peach aphid responses to potato leafroll virus-induced volatiles emitted during disease progression. Environ Entomol. 2009; 38: 1429–1438. 19825298 10.1603/022.038.0511

[33] Ali S, Fathi A. Screening of the susceptibility of newly released genotypes of potato the thrips infestation under field conditions in northwest Iran. Crop Prot. 2014; 62: 79–85

[34] Koschier EH, Sedy KA, Novak J. Influence of plant volatiles on feeding damage caused by the onion thrips *Thrips tabaci*. Crop Prot. 2002; 21: 419–425.

[35] Bockstaller C, Guichard L, Makowski D, Aveline A, Girardin P, Plantureux S. Agri-environmental indicators to assess cropping and farming systems: a review In Sustainable Agriculture 2009 (pp. 725–738). Springer Netherlands.

[36] Cristescu SM, Gietema HA, Blanchet L, Kruitwagen CLJJ, Munnik P, van Klaveren RJ et al Screening for emphysema via exhaled volatile organic compounds. J Breath Res. 2011; 5: 046009 doi: 10.1088/1752-7155/5/4/046009 22071870 10.1088/1752-7155/5/4/046009

[37] Kumar S, Huang J, Abbassi-Ghadi N, Spanel P, Smith D, Hanna GB. Selected ion flow tube mass spectrometry analysis of exhaled breath for volatile organic compound profiling of esophago-gastric cancer. Anal Chem. 2013; 85: 6121–6128. doi: 10.1021/ac4010309 23659180 10.1021/ac4010309

[38] Turechek WW, Hartung JS, McCallister J. Development and optimization of a real-time detection assay for Xanthomonas fragariae in strawberry crown tissue with receiver operating characteristic curve analysis. 2008 Phytopathology 98:359–368. doi: 10.1094/PHYTO-98-3-0359 18944088 10.1094/PHYTO-98-3-0359

[39] van de Wetering F, Hulshof J, Posthuma K, Harrewijn P, Goldbach R, Peters D. Distinct feeding behaviour between sexes of *Frankliniella occidentalis* results in higher scar production and lower tospovirus transmission by females. Entomol Exp Appl. 1998; 88: 9–15.

[40] Stokes ME, Davis CS, Koch GG. Categorical data analysis using SAS. Lane Cove, Australia: SAS institute, 2012.

[41] Robin X, Turck N, Hainard A, Tiberti N, Lisacek F, Sanchez JC et al pROC: an open-source package for R and S plus to analyze and compare ROC curves. BMC Bioinformatics. 2011; 12: 1. 21414208 10.1186/1471-2105-12-77 PMC3068975

[42] Fischer JE, Bachmann LM, Jaeschke R. A readers’ guide to the interpretation of diagnostic test properties: clinical example of sepsis. Intensive Care Med. 2003; 29:1043–1051 doi: 10.1007/s00134-003-1761-8 12734652 10.1007/s00134-003-1761-8

[43] Romeo JT. New SPME guidelines. J Chem Ecol. 2009; 35:1383 doi: 10.1007/s10886-009-9733-2 20054618 10.1007/s10886-009-9733-2

[44] Westmore GC. Thrips vectors and resistance to Tomato spotted wilt virus (TSWV) in potato. Ph.D. Thesis, University of Tasmania. 2012.

[45] Yudin LS, Tabashnik BE, Cho JJ, Mitchell WC. Colonisation of weeds and lettuce by thrips (Thysanoptera: Thripidae). Environ Entomol. 1988; 17: 522–526.

[46] Maharijaya A, Vosman B, Steenhuis-Broers G, Harpenas A, Purwito A, Visser R. G. F. et al Screening of pepper accessions for resistance against two thrips species (*Frankliniella occidentalis* and *Thrips parvispinus*). Euphytica. 2011; 177: 401–410.

[47] Maris PC, Joosten NN, Peters D, Goldbach RW. Thrips resistance in pepper and its consequences for the acquisition and inoculation of tomato spotted wilt virus by the western flower thrips. Phytopathology. 2003; 93: 96–101. doi: 10.1094/PHYTO.2003.93.1.96 18944162 10.1094/PHYTO.2003.93.1.96

[48] Doederlein TA, Sites RW. Host plant preferences of *Frankliniella occidentalis* and *Thrips tabaci* (Thysanoptera: Thripidae) for onions and associated weeds on the southern high plains. J Econ Entomol. 1993; 86: 1706–1713.

[49] Verma SK. Studies on the host preference of the onion thrips, *Thrips tabaci* Lindeman to the varieties of onion. Indian J Entomol. 1966; 28: 396–398.

[50] de Kogel W, van der Hoek M, Mollema C. Oviposition preference of western flower thrips for cucumber leaves from different positions along the plant stem. Entomol Exp Appl. 1997; 82: 283–288.

[51] Broadbent AB, Matteoni JA, Allen WR. Feeding preferences of the western flower thrips, *Frankliniella occidentalis* (Pergande) (Thysanoptera: Thripidae), and incidence of Tomato Spotted Wilt Virus among cultivars of florist’s *Chrysanthemum*. Can Entomol. 1990; 122: 1111–1117.

[52] Herrin B, Warnok D. Resistance of *Impatiens* germplasm to western flower thrips feeding damage. HortSci. 2002; 37: 802–804.

[53] Gaum WG, Giliomee JH, Pringle KL. Resistance of some rose cultivars to the western flower thrips, *Frankliniella occidentalis* (Thysanoptera: Thripidae). Bull Entomol Res. 1994; 84: 487–492.

[54] Nyalugwe E, Wilson CR, Coutts BA, Jones RAC. Biological properties of Potato virus X in potato: effects of mixed infection with Potato virus S and resistance phenotypes in cultivars from three continents. Plant Dis. 2012; 96: 43–54. 10.1094/PDIS-04-11-0305 30731851

[55] Westmore GC, Poke FS, Allen GR, Wilson CR. Genetic and host-associated differentiation within *Thrips tabaci* Lindeman (Thysanoptera: Thripidae) and its links to Tomato spotted wilt virus-vector competence. Heredity. 2013; 111:210–215. doi: 10.1038/hdy.2013.39 23632893 10.1038/hdy.2013.39 PMC3746816

[56] Dickens JC. Sexual maturation and temporal variation of neural responses in adult Colorado potato beetles to volatiles emitted by potato plants. J Chem Ecol. 2000; 26: 1265–1279.

[57] Khalilov LM, Khalilova AZ, Odinokov VN, Baltaev UA, Paramonov EA, Dzhemilev UM. Identification and biological activity of the volatile organic substances emitted by plants and insects II. Sesquiterpenes composition of the native scent of leaves of the potato *Solanum tuberosum*. Chem Nat Compd. 1999; 35: 422–426.

[58] Agelopoulos NG, Pickett J.A. Headspace analysis in chemical ecology: effects of different sampling methods on ratios of volatile compounds present in headspace samples. J Chem Ecol. 1998; 24: 1161–1172.

[59] Agelopoulos NG, Chamberlain K, Pickett JA. Factors affecting volatile emissions of intact potato plants, *Solanum tuberosum*: variability of quantities and stability of ratios. J Chem Ecol. 2000; 26: 497–511.

[60] Webster B, Bruce TJA, Pickett JA, Hardie J. Volatiles functioning as host cues in a blend become nonhost cues when presented alone to the black bean aphid. Anim Behav. 2010; 79: 451–457.

[61] Bruce TJA, Wadhams LJ, Woodcock CM. Insect host location: a volatile situation. Trends Plant Sci. 2005; 10: 269–274. doi: 10.1016/j.tplants.2005.04.003 15949760 10.1016/j.tplants.2005.04.003

[62] Visser JH, Ave DA. General green leaf volatiles in the olfactory orientation of the Colorado Beetle, *Leptinotarsa decemlineata*. Entomol Exp Appl. 1978; 24: 538–549.

[63] Abtew A. Subramanian S, Cheseto X, Kreiter S, Garzia GT, Martin T. Repellency of plant extracts against the legume flowers thrips *Megalurothrips sjostedti* (Thysanoptera: Thripidae). Insects. 2015; 6: 608–625. doi: 10.3390/insects6030608 26463406 10.3390/insects6030608 PMC4598655

[64] Ono M, Igarashi T, Ohno E, Sasaki. Unusual thermal defence against a honeybee against mass attack by hornets. Nature. 1995; 377: 334–335.

[65] Ave DA, Gregory P, Tingey WM. Aphid repellent sesquiterpenes in glandular trichomes of *Solanum bertaultii* and *S*. *tuberosum*. Entomol Exp Appl. 1987; 44: 131–138.

[66] Nault LR, Edwards LJ, Styer WE. Aphid alarm pheromones: secretion and reception. Environ Entomol. 1973; 2: 101–105.

[67] Wohlers P, Tjallingii WF. Electroantennogram responses of aphids to the alarm pheromone (*E*)-β-farnesene. Ent Exp Appl. 1983; 33: 79–82

[68] Koschier EH. de Kogel WJ Visser JH. Assessing the attractiveness of volatile plant compounds to western flower thrips *Frankliniella occidentalis*. J Chem Ecol. 2000; 26: 2643–2655.

[69] Weissbecker B, van Loon JJA, Posthumus MA, Boumeester HJ. Dicke M. Identification of volatile potato sesquiterpenoid and their olfactory detection by the two-spotted stinkbug *Perillus bioculatus*. J ChemEcol. 2000; 26: 1433–1445.

[70] Cook SM, Khan ZR, Pickett JA. The use of push-pull strategies in integrated pest management. Ann Rev Entomol. 2006; 52: 375–400. 10.1146/annurev.ento.52.110405.091407 16968206

[71] van Toi RWHM, James DE, de Kogel WJ, Teulon DAJ. Plant odours with potential for a push-pull strategy to control the onion thrips, Thrips tabaci. Entomol Exp Appl. 2007; 122: 69–76.

