# Supplementary material for: Receiver Operating Characteristic curve analysis determines association of individual potato foliage volatiles with onion thrips preference, cultivar and plant age
Source: PLoS One. 2017 Jul 26;12(7):e0181831. doi: 10.1371/journal.pone.0181831 (PMC5528874; doi:10.1371/journal.pone.0181831)
Supplement: S1 Table — (DOCX) [file pone.0181831.s001.docx]

**S1 Table. Experimental details for the thrips preference trials**

|  | **Paired plant choice experiments** | |  | **Five plant preference experiments** | |
| --- | --- | --- | --- | --- | --- |
|  | **Access** | **No Access** |  | **Expt 1** | **Expt 2** |
| Cultivar pairs or cultivars tested | 4 | 4 |  | 5 | 5 |
| Sample times | 8 | 6 |  | 1 | 1 |
| Replicates | 6 | 6 |  | 6 | 6 |
| Thrips added to chamber per replicate | 25 | 25 |  | 100-150 | 100-150 |
| Thrips counted per replicate:  mean (range) | 9.3 (5-16) | 10.1 (5-18) |  | 31.7 (15-68) | 19.8 (12-29) |
| Thrips counted per experiment | 224 | 243 |  | 190 | 119 |
